# Prevalence of Maltreatment Among Canadian National Team Athletes

**DOI:** 10.1177/08862605211045096

**Published:** 2021-09-22

**Authors:** Erin Willson, Gretchen Kerr, Ashley Stirling, Stephanie Buono

**Affiliations:** University of Toronto, ON, Canada

**Keywords:** violence exposure, youth violence, child abuse

## Abstract

This study assessed the prevalence of maltreatment experienced by Canadian National Team athletes. In total, 995 athletes participated in this study, including current athletes and athletes who had retired in the past 10 years. An anonymous online survey was administered, consisting of questions about experiences of psychological, physical, and sexual harm, and neglect, as well as questions about identity characteristics, when the harm was experienced, and who perpetrated the harm. Neglect and psychological harm were most frequently reported, followed by sexual harm and physical harm. Female athletes reported significantly more experiences of all forms of harm. Retired athletes reported significantly more neglect and physical harm. Athletes reportedly experienced more harmful behaviors during their time on the national team than before joining a national team. Coaches were the most common perpetrators of all harms except for sexual harm, which was most frequently perpetrated by peers. This study highlighted the prevalence with which Canadian National Team athletes reportedly experience harmful behaviors in sport, suggesting the need for preventative and intervention initiatives.

## Introduction

Despite high-profile and disturbing cases of athlete maltreatment, such as the case of Nassar, a USA Gymnastics team doctor who was convicted of sexually assaulting over 150 minors (Levinson, 2018), empirical work on the maltreatment of athletes is in its infancy. Research on sexual abuse of athletes began a few decades ago and empirical work on psychological abuse is emerging but research on physical abuse and neglect is lacking. Researchers have proposed that the lack of empirical work on athlete maltreatment is attributable to assumptions of sport as a moral oasis (Brackenridge, 2001) or the assumed essential goodness and purity of sport (Coakley, 2011). Given the lack of research on athlete maltreatment, questions about prevalence, the nature of athletes’ experiences, and effective prevention and intervention initiatives remain. This study aims to contribute to this body of knowledge by exploring prevalence rates of athlete maltreatment.

Maltreatment is understood as an umbrella term encompassing “…all types of physical and/or emotional ill-treatment, sexual abuse, neglect, negligence, and commercial, or other exploitation, which results in actual or potential harm to the child’s health, survival, development, or dignity in the context of a relationship of responsibility, trust, or power (World Health Organization, 2020). Maltreatment can be divided into relational and nonrelational categories, based upon the nature of the relationship in which the behavior occurs. Relational maltreatment exists within a critical relationship, where one actor has significant influence on another’s sense of trust, security, or fulfilment of needs (Crooks & Wolfe, 2007), such as the relationship between a parent and child, a teacher and student, or a coach and their athlete. Relational maltreatment includes sexual, physical and psychological abuse, and neglect. Examples of sexual abuse in sport include sexual relationships between a coach and underaged athlete, and inappropriate touching (Stirling, 2009). Psychological abuse in sport can include: verbal behaviors, such as repeated demeaning or humiliating comments; physical behaviors, including throwing objects; and denial of attention or support, such as intentionally ignoring an athlete for poor performance (Stirling & Kerr, 2008). Physical abuse in sport can include contact behaviors such as striking an athlete or noncontact behaviors such as administering exercise as punishment (Stirling, 2009). Neglectful behaviors are omissions of care and in sport can be exhibited by failing to provide adequate injury care or supervision (Stirling, 2009). In contrast, nonrelational maltreatment, which does not occur within a critical relationship, includes harassment (e.g., derogatory comments by a superior), and bullying (e.g., spreading rumors about teammates) (Stirling, 2009).

To understand the prevalence of maltreatment in sport, national scale studies have been conducted in the United Kingdom (Alexander et al., 2011) and in Belgium and the Netherlands (Vertommen et al., 2016). In both studies, retrospective surveys that asked adults (over the age of 18 years) to reflect on their childhood experiences of sport revealed that psychological harm was the most frequently reported form of harm experienced, followed by sexual harm and physical harm (Alexander et al., 2011; Vertommen et al., 2016). Neither study assessed experiences of neglect. Additionally, both studies found that athletes at higher levels of competition (i.e., national and international) reported higher rates of maltreatment than athletes competing at lower levels. Vertommen et al. (2016), who used the term “interpersonal violence” rather than maltreatment, also found sexual orientation was a risk factor for sexual, physical, and psychological violence, while gender and ethnicity were significant risk factors for physical and sexual violence, and having a disability was a risk factor for sexual violence.

In Canada, the last national prevalence study was conducted in 1996 (Kirby & Greaves, 1996), focused primarily on sexual abuse and did not explore identity characteristics such as race or sexual orientation. Since that time, societal awareness of sexual violence has increased substantially through such movements as #MeToo (Alaggia & Wang, 2020; Rotenberg & Cotter, 2018). Additionally, our empirical understanding of maltreatment in sport has grown, particularly with respect to nonsexual forms of maltreatment. We have come to understand that those with underrepresented identities have increased risk and actual experiences of violence (Status of Women, 2020), and data regarding experiences of maltreatment in sport by athletes from these groups are lacking. While the coach-athlete relationship has been the primary focus of the maltreatment in sport literature (Burke, 2001; Gervis & Dunn, 2004; Stirling & Kerr, 2009), research is lacking on other potential perpetrators such as sport administrators and support staff and yet, as the Larry Nassar case highlighted (Levinson, 2018), other members of the sport community can be perpetrators. There have also been several initiatives in Canada that have targeted the prevention of maltreatment in sport. For example, the Responsible Coaching Movement in Canada advocates for the “Rule of Two” to prevent incidences of maltreatment (Coaching Association of Canada, n.d.). The “Rule of Two,” which recommends that two certified coaches are present at all times. This may help to prevent sexual harms, which typically occur in private, but does not address psychological harms which are normalized in sport and occur in public (Kerr et al., 2016). Understanding where the problems lie in terms of types and rates of harms will help to inform evidence-based policy and other prevention and intervention initiatives, and will provide a baseline against which to assess the impact of future initiatives.

Therefore, the purpose of this study was to assess the prevalence of maltreatment among current and former National Team athletes in Canada. More specifically, this study sought to examine the following: (a) the prevalence of maltreatment (physical, sexual, psychological harm, and neglect); (b) when the harm occurred (i.e., before or during membership on the National Team); (c) whether identity characteristics (e.g., race, gender, sexuality, disability) affect reported experiences of maltreatment; and (d) the perpetrators of maltreatment in sport.

## Methods

### Participants

Participants in this study were current and retired Canadian National Team athletes, including para and non-para-athletes. All participants were required to be over the age of 16 years and could be of any gender. Retired athletes were included only if they had retired within the previous 10 years, to help protect against limitations of memory recall. Athletes from any sport in Canada that had a national team were welcome to participate. The distinction to include national team athletes rather than Olympic sports was intentional to expand reach, given that several sports have national teams that do not compete in the Olympic Games. The national team population was selected because it is the highest level of competition in Canada and previous research in other countries has revealed a higher prevalence of all forms of maltreatment at this level compared to lower levels of competitive sport (Brackenridge, 2001; Gervis & Dunn, 2004).

### Recruitment

After receiving ethical approval from the University of Toronto for this study, recruitment occurred through an association named AthletesCAN which represents Canadian National Team athletes and whose mission is to “be the collective voice for Canadian Athletes and to empower athletes to achieve their full potential inside and outside of sport” (AthletesCAN, n.d.). AthletesCAN maintains a membership database of current and retired Canadian National Team athletes along with their contact information. AthletesCAN agreed to assist by distributing an email that included an invitation to participate, a letter of information that specified the inclusion criteria, and links to the survey through their membership listserv, which consisted of 6,239 Canadian National Team athletes who were competing at that time or had competed for Canada in the prior ten years. AthletesCAN also used the social media platforms of Instagram and Facebook to recruit. Potential participants were informed of the purpose of the study, given assurance that the survey would be completed anonymously, that participation was voluntary, and that only aggregate data would be presented. The survey was open for 30 days from March 12th to April 11th, 2019, and two reminders for completion were sent during this time. There was no compensation provided for completing this survey.

### Measures

An anonymous, online survey was used to measure experiences of athlete maltreatment. There were 121 questions in this survey that assessed various topics of interest including identity characteristics (age, sport, whether the athlete identified as male/female/other, LGBTQ2I+, Indigenous, having a disability, and/or racialized, athlete satisfaction, maltreatment experiences, perceived outcomes of maltreatment experiences, and disclosure and reporting of these experiences. However, for the purposes of this article and answering the specific research questions pertaining to identity characteristics and prevalence, a subset of items (*n* = 39) was used, including 31 maltreatment-related items and 8 identity-related items.

The maltreatment-related items asked about athletes’ experiences with relational (sexual abuse, physical abuse, psychological abuse, and neglect) and nonrelational maltreatment (bullying, harassment, discrimination), so the term “harm” was adopted to encompass objective behaviors in both categories. Questions began with the prompt “Think of your experiences with coaches, parents, teammates or peers in sport, trainers, sport administrators, high performance director, and strangers during your national team experience.” Questions about neglect referred to such experiences as training in unsafe conditions and lack of attention to educational needs. Questions about psychological harm included “you were put down, embarrassed or humiliated,” and “you have been criticized as a person when your performance was subpar.” Sample questions about physical harm included “you have been punished with excessive exercise” and “you have been punched/hit,” and questions about sexual harm included “you were the target of sexist jokes/remarks” and “you have been touched in sexually inappropriate ways.”

The eight neglect items had moderate internal consistency (α = .67); the nine psychological items had high internal consistency (α = .84); the nine sexual harm items had moderate internal consistency (α = .76), and the five physical harm items had low internal consistency (α = .32). Questions addressing “when” and “by whom” harm occurred were structured so that more than one answer could be selected if the athlete had experienced a particular behavior at multiple time points or by multiple perpetrators. If participants answered “yes” to experiencing a behavior, follow-up questions were posed about when the maltreatment occurred (i.e., prior to or during the national team career) and who perpetrated the maltreatment (choices included: coach, trainer, peer or teammate, parent, sport administrator, high performance director, stranger). If the participant answered “no” to experiencing a behavior, the next question was presented. For the analysis, we used mean scores for perpetrators and the time periods.

The survey was designed for the purposes of this study given the absence of a validated scale of athlete maltreatment. The development of the survey items was informed by the literature and in partnership with AthletesCAN’s Safe Sport working group of 8 members, including male and female para and non-para-athletes, from a variety of individual and team sports. The diverse membership of this working group was crucial for ensuring that the survey questions were relevant to various athletes and sports. The survey included questions used in Vertommen et al.’s (2016) study, behaviors that Stirling (2009) identified as maltreatment, and questions recommended by AthletesCAN.

Four versions of the survey were available, including one each for current and retired athletes, with both available in French and English, the two official languages in Canada. Links to the French and English surveys were provided so that participants could choose which language they preferred. Participants indicated whether they were a current or retired athlete and the corresponding survey was provided. The current and retired versions differed in tense use, for example, “You have been pressured…” for the current athletes and “You were pressured…” for the retired athletes. The survey was completed anonymously, and participants were assured that only aggregate data would be reported.

### Procedure

The initial version of the survey was sent to AthletesCAN, its Safe Sport working group and select athletes to pilot the relevance and comprehension of the questions, and to check the time required to complete the survey. Revisions were made based upon the pilot to ensure the use of inclusive language (i.e., changing from LGBTQ+ to LGBTQ2I+), as well as clarity, tone, and relevance of the questions. Also, emotional responses to behaviors such as “that made you uncomfortable” were removed from questions such as “you have been looked at with a sexually intrusive glance” to ensure a focus on objective behaviors only. The survey was then uploaded on the Research Electronic Data Capture (REDCap), which is a secure web application through the University of Toronto. The survey required approximately 15 minutes to complete.

The submitted surveys were routed directly to the researchers for analysis and stored on a password protected and encrypted computer. Participants who retired outside of the 10-year maximum were removed from the data set and were not included in the analysis. Incomplete surveys were included in the analysis. Due to the sensitive nature of the questions, athletes may have chosen to skip some questions or not finish the survey, but we thought it was important to include the responses that were provided. Anonymity and confidentiality were expressed concerns of AthletesCAN due to the sensitive nature of the topic and fear of repercussions from their sport organizations. No names were collected, the letter of information stated that only aggregated results would be reported and that sport organizations would not have any knowledge of their participation. To ensure confidentiality of the participants, we did not analyze the data by sport. Given the small pool of National Team athletes in some sports and the 10-year time span, it would be possible for sport organizations to identify athletes who completed the survey.

### Data Collection and Analysis

#### Research question 1: What is the prevalence of maltreatment?

The first research question addressed the prevalence of maltreatment among Canadian National Team Athletes. Prevalence was calculated in two ways; first, by calculating the frequency with which *athletes* reported at least one experience of each form of maltreatment, a calculation used in previous prevalence studies (Vertommen et al., 2016). For example, 551 athletes responded “yes” and 250 athletes responded “no” to experiencing at least one behavior within the neglect category; therefore, of 801 respondents, 69% reportedly experienced at least one neglectful behavior. Secondly, the frequency of harmful behaviors experienced within each category of harm (i.e., neglect, psychological, physical, sexual), was calculated, that is, the total number of times athletes responded “yes” to experiencing harmful behaviors out of the total number of possible harmful behaviors experienced per category of harm. This analysis accounted for the different number of questions within each category. Finally, a correlation matrix analysis was conducted to assess potential relationships between the forms of harm.

#### Research question 2: When does harm occur?

The second research question addressed in this study was, when are athletes more likely to experience harm, before or during their time as a national team member? Given the focus of interest on when the maltreatment experiences occurred, current and retired athletes were analyzed together. A *t*-test was used to analyze whether athletes were significantly more likely to experience harm before or during their national team experience.

#### Research question 3: Who experiences harm?

The third research question addressed was whether athletes with certain identity factors (e.g., racialized, athletes with a disability) were more likely to experience harm? A logistic regression analysis was conducted to address this research question, and unadjusted odds ratios were used to compare identity factors (i.e., gender, racialized, Indigenous, LGBTQ2I+, disability). Athlete identity factors were analyzed as predictors with outcomes of experiences of harm. All athletes except one identified as either male or female; the data for this athlete was removed for the gender analysis. Chi-squared analysis was used to compare prevalence rates for current and retired athletes. The chi-squared matrix analyzed the proportion of current athletes (current = yes) and retired athletes (retired = yes) by incidence of all forms of harm (e.g., psychological harm = yes).

#### Research question 4: Who are the perpetrators of harm?

The fourth research question addressed was, who were the most commonly reported perpetrators of harm? Means and standard deviations were used to identify perpetrators.

## Results

### Participants

There were 1,001 surveys returned although 6 of these were blank, resulting in 995 respondents, including 758 current and 237 retired athletes, comprising a 16% response rate. The mean age of participants was 27.7 years (*SD* = 9.09): 25.5 years (*SD* = 8.16) for current and 34.4 years (*SD* = 8.57) for retired athletes. Of the respondents, 61.5% identified as females, 38.4% identified as males, and .1% identified as other. Participants self-identified as racialized (9.7%), having a disability (11.6%), Indigenous (1.6%), and LGBTQ2I+ (7.3%). The total number of respondents differed across the demographic results because not all participants responded to each question. Respondents represented 64 sports. The sports with the highest participation rates were gymnastics (5.5%), volleyball (5.4%), athletics (4.4%), swimming (3.8%), rowing (3.3%), rugby (3.5%), hockey (3.4%), and freestyle skiing (3.8%).

### Missing Data

A sensitivity analysis was conducted to examine potential differences in participants who completed the entirety of the questions in each category of harm versus participants who did not complete each category of questions. The neglect scale was answered by 80.5% (*n* = 801) of participants. There were 44 participants who answered some items but did not complete the entire scale. The psychological harm scale was answered by 80% (*n* = 794) of participants; 23 participants answered some items but did not complete the entire scale. The physical harm scale was answered by 79.6% (*n* = 792) of participants; there were 8 participants who answered some items but did not complete the entire scale. The sexual harm scale was answered by 80% (*n* = 794) of participants; 15 participants answered some items but did not complete the entire scale.

The sensitivity analysis revealed a significant difference in reports of neglect with participants who completed the survey reporting higher average neglect scores than participants who did not (*t* = –3.32, *p* = .002). No significant differences existed in reports of psychological harm (*t* = .32, *p* = .75), physical harm (*t* = .92, *p* = .39), and sexual harm (*t* = .98, *p* = .34) between participants who did and did not complete these respective scales.

### What is the Prevalence of Maltreatment?

Regarding the first question on the prevalence of maltreatment, overall, 75% (*n* = 751) of the 995 athletes responded “yes” to experiencing *at least one* potentially harmful behavior across all categories of harm (i.e., physical, psychological, sexual, and neglect). The highest proportion of athletes (68.8%; *n* = 551) reported experiencing *at least one* neglectful behavior, followed by 60.2% (*n* = 478) of athletes reporting *at least one* psychological harmful behavior. *At least one* sexually harmful behavior was reported by 20.5% (*n* = 163) of athletes and *at least one* physically harmful behavior was reportedly experienced by 14.3% (*n* = 113) athletes.

In addition to examining the percentage of athletes who reported experiencing at least one harmful behavior in each category, the percentage of reported behaviors within each type of harm was also examined (i.e., number of harmful behaviors reported/total possible number of harmful behaviors within each type of harm). The most common form of harmful behavior was psychological, as athletes reportedly experienced an average of 2.6 psychologically harmful behaviors of a possible 9 (24%). Second, athletes reported experiencing an average of 3.4 neglectful behaviors of a possible 7 (23.7%). Far fewer sexually harmful behaviors (4.7%), and physically harmful behaviors were reportedly experienced (3.4%). The most frequently reported behaviors within each category of harm are identified in the following sections.

#### Neglect.

Table 1 indicates that the most commonly experienced behaviors of neglect as reported by the current and retired athletes were training while injured or exhausted, followed by neglect of career and/or educational needs, and being generally ignored.

#### Psychological harm.

The most commonly reported behaviors of psychological harm were being shouted at in an angry or critical manner, being gossiped about, having lies told about the individual, being put down, embarrassed or humiliated, being intentionally ignored in response to poor performance, and criticized as a person (Table 1).

#### Sexual harm.

Sexually harmful behaviors that were most commonly reported included experiencing sexist jokes and remarks, intrusive sexual glances, sexually explicit communication, and sexually inappropriate touching (Table 1).

**Table 1.** Percent of Athletes Who Reported Experiences of Harmful Behaviors.

	% of Athletes Reporting At Least One Experience
**Neglect**	
Training when injured/exhausted	30.1
Sacrificed career/education	27.7
Felt generally ignored	23.2
Trained/competed in unsafe conditions	15.2
Inadequate attention to psychological readiness in trying new skills	9.8
Inadequate support of basic needs	8.9
Inappropriately left alone with no care	6.7
**Psychological harm**	
Shouted at in an angry or critical manner	33.3
People have gossiped or told lies about you	30.1
Put down, embarrassed, or humiliated	27.9
Intentionally ignored in response to poor performance	27.6
Criticized as a person for subpar performance	23.9
Removed (or threats of removal) from practice or team	20.1
Negatively criticized about your body or weight	19.8
Called names or otherwise offended	17.3
Sworn/cursed at for not performing well	16.1
**Sexual harm**	
Sexist jokes/remarks	14.9
Intrusive sexual glances	9.2
Sexually inappropriate touching	4
Sexually explicit communication	4
Someone has tried to have sex with you against your will	3.2
Someone has exposed him/herself to you	2.8
Sex with penetration against your will	1.6
Made to kiss someone against your will	1.4
Asked to undress, assume a sexually explicit pose	1.3
**Physical harm**	
Punished with excessive exercise	12.9
Slapped/hit with an open hand	1.4
Hit with an object	1.4
Forced to the ground/knocked down	1
Punched/hit with a fist	0.4

#### Physical harm.

The most commonly reported physically harmful behavior was being punished with excessive exercise (Table 1). Other forms of physical harm were rarely reported.

#### Correlations between forms of harm.

Table 2 demonstrates that each form of harm had a significant positive relationship with the other forms, indicating that athletes who experience harm likely experience more than one form of harm in their training and competition environments.

### When Do Experiences of Harm Occur?

The second research question addressed whether athletes reported more experiences of harm before or during their time on a national team. The results indicated that the athletes reported significantly more experiences of each form of harm (neglect [*t*(525) = 21.02, *p* < .0001], psychological [*t*(477) = 13.52, *p* < .0001], sexual [*t*(162) = 5.28, *p* < .0001], and physical [*t*(277) = 9.96, *p* < .0001]) when they were on a national team compared to their athletic career before joining a national team.

### Who Experiences Harm?

The third research question was whether experiences of harm differed according to identity characteristics of the athletes. Chi-squared analysis indicated that retired athletes reported significantly more experiences of neglect, (χ2 [1, 801] = 6.97, *p =* .008) and physical harm (χ2 [1, 792] = 5.8, *p =* .019) compared to current athletes, but there were no significant differences between current and retired athletes for psychological (χ2 [1, 794] = 1.31, *p =* .163) and sexual (χ2 [1, 794] = .84, *p =* .365) harm.

**Table 2.** Correlations Between Types of Harm.

Correlations Coefficients for Types of Harm (Current and Retired Athletes Combined)				
	Neglect	Psychological Harm	Physical Harm	Sexual Harm
Neglect		.498**	.305**	.503**
Psychological harm			.406**	.989**
Physical harm				.408**
Sexual harm				

*Note*. **Correlation is significant at the 0.01 level (two-tailed).

**Table 3.** Unadjusted Odds Ratio of Predicting Harm From Identity Factors.

	Neglect	Psychological Harm	Sexual Harm	Physical Harm
Gender	2.490**	2.524**	6.755**	1.592*
Race	1.286	.845	1.514	2.596*
Disability	.805	.700	.809	.516
Indigenous	1.022	.290*	.702	.501
LGBTQ2I+	1.665	1.108	2.269*	.917

*Note.* *indicates significance at .05.

**indicates significance at .001.

Characteristics of gender, race, sexual orientation, Indigeneity, and disability status were also examined. Logistic regressions indicated that female athletes experienced significantly higher rates of all forms of harm than male athletes (see Table 3 for unadjusted odds ratios). Athletes who identified as Indigenous experienced significantly lower rates of psychological harm than non-Indigenous athletes; athletes who identified as racialized experienced significantly higher rates of physical harm than nonracialized athletes; and LGBTQ2I+ identifying athletes experienced significantly more sexual harm than non-LGBTQ2I+ athletes. There were no significant differences in reported harms between athletes with or without a disability (Table 3).

### Perpetrators of Harm

To address the fourth research question, athletes’ responses to the perpetrators of harmful behaviors were examined. For neglect, coaches (*M =* 1.66, *SD* = 1.29) were the most frequently identified perpetrator followed by high-performance directors (*M* = 1.10, *SD* = 1.12), sport administrators (*M* = .91, *SD* = 1.10), peers (*M =* 51, *SD* = .03), trainers (*M* = .45, *SD* = 1.29), others (*M* = .24, *SD* = .03), parents (*M* = .19, *SD* = .02), and strangers (*M* = .18, *SD* = .02). For psychological harm, the perpetrators of harm, in descending order, were coaches (*M* = 2.66, *SD* = 2.46), peers (*M* = 1.04, *SD* = 1.37), high-performance directors (*M* = 0.64, *SD* = 1.30), sport administrators (*M* = .41, *SD* = 1.05), trainers (*M =* .29, *SD =* .04), strangers (*M =* .26, *SD =* 03), parents (*M =* .19, *SD =* .03), and others (*M =* .18, *SD =* .03). For sexual harm, peers (*M* = .91, *SD* = 1.22) were identified as the most frequent perpetrators, followed closely by coaches (*M* = .84, *SD* = 1.24), then strangers (*M* = .59, *SD* = 1.08), others (*M* = .36, *SD* = .87), trainers (*M =* .15, *SD =* .04), sport administrators (*M =* .13, *SD =* .03), high-performance directors (*M =* .09, *SD =* 0.2), and parents (*M =* .05, *SD =* .02). The most frequently identified perpetrator for physical harm was coaches (*M* = 1.14, *SD* = .62), followed by trainers (*M* = .31, *SD* = .55), high-performance directors (*M =* 20, *SD =* .03), peers (*M* = .18, *SD* = .40), sport administrators (*M =* .09, *SD =* .02), parents (*M* = .07, *SD* = .36), others (*M* = .05*, SD =* .01), and strangers (*M =* .03, *SD =* .02).

## Discussion

This study assessed the prevalence of maltreatment experienced by Canadian National Team athletes, including various forms of harm, influences of athletes’ identities on experiences of maltreatment, and the timing and perpetrators of harm. Overall, a high proportion of athletes (75%) reportedly experienced at least one harmful behavior in the sport context.

Results from this study indicated that psychological harm and neglect were the most frequently experienced forms of maltreatment; to a much lesser extent, sexual, and physical harm were reported. The rank ordering of psychological, sexual, and physical harm is consistent with the findings of previous prevalence studies of maltreatment in sport (Alexander et al., 2011; Vertommen et al., 2016), however, previous studies have not included assessments of neglect. The current study highlights the importance of considering neglectful behaviors in sport, particularly given the documented negative impacts of neglect on health and well-being (Hildyard & Wolfe, 2002).

Psychological harm was also a frequently reported type of harm in this study, confirming the findings of other prevalence studies in sport (Alexander et al., 2011; Vertommen et al., 2016). The prevalence of psychologically harmful behaviors in sport has been previously attributed to the normalization of these behaviors (Smits et al., 2017; Stirling & Kerr, 2008), and assumptions that psychologically abusive coaching methods are necessary to develop talent in the sport context (Stafford et al., 2015; Stirling & Kerr, 2009). Given the well-documented short and long-term negative outcomes associated with experiences of psychological abuse (Arata et al., 2005; Kent & Waller, 2000), future research on alternative coaching strategies is needed.

While sexual harm was reportedly experienced at lower rates (5%) than psychological harm and neglect, 20% of the athletes in the current study experienced at least one harmful behavior in this category. This rate is slightly higher than results of other studies on sexual violence in sport (Fasting et al., 2003; Leahy et al., 2002; Vertommen et al., 2016) perhaps due to the inclusion of both contact and noncontact forms of sexual harm in the current study. Frequently reported noncontact behaviors in the current study such as sexist jokes and remarks, intrusive sexual glances, and sexually explicit communication demonstrates that a culture of sexual violence persists through verbal and nonverbal communication. The higher prevalence of reported sexual harm in this study may also be attributable to the recent cultural shift and increased awareness of sexual violence through campaigns such as #MeToo.

Physically harmful behaviors were reportedly experienced at lower rates than the other forms of harm. The regulations of physically harmful behaviors in sport and society may account for this finding as contact forms, such as being hit with an object or being slapped, are easy to observe, report, and sanction. However, the most frequently reported physically harmful behavior in the current study was a noncontact form of using exercise as punishment. This was a novel aspect explored in this study that was not examined in previous prevalence studies (Alexander et al., 2011; Vertommen et al., 2016) and highlights the potential harm associated with this commonly used sport practice. However, given the low internal consistency value associated with the items in the physical harm scale, further work is needed in this area.

Importantly, the findings of this study indicate significant positive correlations between the various forms of harm, suggesting that an environment that is conducive to one form of harm is likely conducive to many forms of harm. Together, the findings of the current study and the extensive body of literature noting the lack of power and autonomy experienced by athletes (Potrac et al., 2002; Rylander, 2016), suggest that the characteristics of the sport environment that leave athletes vulnerable to potentially harmful experiences need further attention.

Retired athletes reported significantly higher rates of neglect and physical harm than current athletes. While these findings could indicate that the maltreatment experiences of national team members have decreased recently, we posit an alternative explanation. Evidence exists that indicates that time and distance from the sport environment can shift athletes’ perspectives of their sport experiences. For example, behaviors that athletes classified as normal and were accepted during their careers have been shown to be relabeled as abusive and harmful once they leave the sport (Stafford et al., 2015). Given that potentially harmful behaviors in sport are normalized, we posit that athletes learn to accept inappropriate behaviors during their careers but, upon reflection in retirement, reappraise these experiences as harmful.

Additionally, in all categories of harm, behaviors were more frequently reported to have occurred during, rather than prior to, the national team experience. This is consistent with previous findings demonstrating that athletes at higher levels of sport competition (e.g., competing at an international level) have increased prevalence rates of harm compared to athletes at other levels of sport (Alexander et al., 2011; Gervis et al., 2016; Vertommen et al., 2016). These findings reinforce the need for protection of athletes at high levels of sport. Additionally, many athletes are competing on national teams during their adult years, which indicates the need to extend the focus on maltreatment and safeguarding beyond child or youth populations, to all athletes competing in sport, regardless of age.

With respect to the findings regarding gender, female athletes reported significantly higher rates of all forms of maltreatment compared to males, supporting existing gender-based and interpersonal violence research outside of sport (Balsam et al., 2005; Friedman et al., 2011; Status of Women, 2020). However, in contrast to the current findings, previous sport prevalence studies did not find any gender differences in psychological harm (Alexander et al., 2011; Vertommen et al., 2016), potentially due to differences in samples between the studies. Also, Vertommen et al. (2016) reported that male athletes reported higher rates of physical harm than female athletes, in contrast to the findings of the current study. This difference could be attributed to the inclusion of exercise as punishment in the current study, which was the most frequently reported form of physical harm, and was not included as an item in previous studies.

Athletes who identified as LGBTQ2I+ reported significantly more experiences of sexual harm than non-LGBTQ2I+ identifying athletes. This finding is consistent with previous studies (Vertommen et al., 2016), and the general sexual abuse literature (Katz-Wise & Hyde, 2012). Additionally, athletes who identified as racialized experienced significantly higher rates of physical harm than nonidentifying athletes, similar to Vertommen et al. (2016) finding that being of an ethnic minority was a risk factor for physical harm. Physical violence toward racialized individuals is a concern in North American society, with continued reports of violence toward Black and Asian populations in particular (Joseph, 2021); the current study’s finding may indicate there are similar trends in sport. The finding that psychological harm was significantly lower for athletes who identified as Indigenous is difficult to interpret but raises questions about the low sample size and possible interactions with gender and sport type. In contrast to research in the general population indicating that individuals with a disability experience more abuse than individuals without a disability (Horner-Johnson & Drum, 2006; Sullivan & Knutson, 2000), there were no significant differences in experiences of harm between athletes with or without a disability. Given the existing research demonstrating the increased risk of violence experienced by those with underrepresented identities (Turell et al., 2018), future research is needed on the experiences of athletes with underrepresented identities.

Coaches were the most frequently reported perpetrator of psychological and physical harm, and neglect, while sexual harm was most frequently perpetrated by peers. Research on sexual harm in sport focuses primarily on the coach-athlete relationship (Brackenridge et al., 2008; Burke, 2001; Kirby et al., 2000), but our findings suggest that other relationships need to be explored further. High-performance directors, sport administrators, trainers, and peers, were cited as actors of harm, consistent with identified perpetrators of maltreatment in previous studies (Alexander et al., 2011; Vertommen et al., 2016). The findings regarding the various groups of perpetrators suggest there are aspects of the sport context that enable, encourage and/or normalize potentially harmful behaviors and thus, greater attention needs to be devoted to the entire ecosystem of sport.

### Limitations

The sample of this study had a low proportion of respondents who self-identified as racialized, Indigenous, LGBTQ2+, or with a disability. However, the lack of diversity within the sample is characteristic of the Canadian National Team population which does not mirror the diversity seen in the general population (Lawrence, 2017). Further, it is not known whether these findings are generalizable to athletes at other levels of sport or whether athletes leave sport prior to the National level as a result of experiences of maltreatment.

The study had a response rate of 16%. Given that the sample included athletes who had retired over the previous 10 years, athletes’ contact information in AthletesCAN’s database may not have been accurate. The subject matter of maltreatment and the sensitive nature of the survey questions may have influenced the response rate, either by deterring participation of those who have been negatively affected by maltreatment experiences and found it too psychologically difficult to participate, or by attracting participation by those who had experienced maltreatment more so than for those without these experiences. The study is therefore limited by the inability to compare the characteristics and experiences of those who did and did not participate. As the total survey consisted of 121 items, the length of the survey could potentially have been a deterrent to participation or may have contributed to participants starting but not completing the survey. It is possible that response patterns are related to survey completion for some forms of harm given the differences in reports of neglect, but not in reports of other harms, between participants who completed the entire scale versus participants who did not. The findings are also limited by the lack of a psychometrically validated scale of athlete maltreatment and the low internal consistency of the physical harm scale.

### Implications

As the first prevalence study of all forms of maltreatment within a Canadian sample of elite athletes, the findings indicate that maltreatment characterizes the sport experience for many. The predominance of reported experiences of psychological harm and neglect suggest that intervention strategies should extend beyond the current focus on prevention of sexual harms within the coaching community to consider all forms of maltreatment. Further, the normalization of some disconcerting behaviors (e.g., yelling, use of exercise as punishment) in sport may call for different intervention strategies. As the most frequent perpetrators of harm were coaches, implications may be drawn for more coaching education with a focus on developmental psychology and evidence-based teaching and learning methods. Given that other stakeholders in sport were also identified as perpetrators of harm, future researchers should extend the focus to the nature of other relationships athletes have in the sport context. As this study focused on athletes at the highest level of sport, future studies should investigate athletes’ experiences at other levels of sport, including grassroots, community sport, school sport, and club-based sport.

The data also highlight the differential experiences of those athletes with specific identity characteristics. Although the proportion of athletes with underrepresented identities in this study was far less than in the general Canadian society—a finding in and of itself—the data suggest that athletes who identify as racialized, Indigenous or sexually diverse, have different experiences of maltreatment. The findings have implications for further study of power imbalances that foster or enable maltreatment of athletes within key sporting relationships. This investigation of power influences should extend to athletes in emerging adulthood and adulthood given the finding that athletes over the age of 16 reportedly experienced various harms.

Future research in sport would benefit from the development of a standardized, psychometrically reliable, and valid measure to assess the prevalence of maltreatment in sport. Further, prospective, repeated measures, and longitudinal research designs are recommended to ascertain changes in experiences over time and across an athletic career. Such designs may also enable the assessment of the impact or effectiveness of future preventative and intervention initiatives. From an applied perspective, the current findings regarding experiences of various harms suggest that future research and applied work should address ways in which the elite sport environment can be more positive, free from harms, and exemplify evidence-based methods of coaching to develop talent in young people.

### Conclusion

This study indicated the prevalence with which Canadian National Team athletes reportedly experienced various forms of harm. The findings reinforce those of previous prevalence studies that highlight the predominance of psychologically harmful experiences in sport and the need for a targeted approach to dismantle the normalization of these behaviors. A novel contribution of this study to the body of work on athlete maltreatment is the prevalence of experiences of neglect—an area in need of further study and inclusion in policies and educational initiatives. The athletes reported more experiences of harm during their National Team careers than prior to joining the National Team suggesting that power imbalances extend to adult athletes. Coaches were the most common perpetrator of all harms except for sexual harm which was most frequently perpetrated by peers, potentially suggesting the need for different prevention or intervention strategies for different forms of harm. This study contributes to our understanding of the prevalence of maltreatment experiences within a specific sport context and indicates a need for more empirical attention on the nature of experiences of athletes of varying identities, levels of sport, characteristics of the sport environment that leave athletes vulnerable to potentially harmful experiences, and the development of prevention and intervention measures for all athletes regardless of age.

## Author Biographies

**Erin Willson**, MSc, is currently a PhD student in the Faculty of Kinesiology and Physical Education at the University of Toronto. Her areas of research interest include maltreatment in sport, athlete empowerment and advocacy. As a former Olympian, Erin brings a unique perspective to her research endeavors.

**Gretchen Kerr**, PhD, is a professor and Dean of Faculty of Kinesiology and Physical Education and vice-dean of the School of Graduate Studies, at the University of Toronto. Her research focuses on the psychosocial health of young people in elite sport, athlete maltreatment, and the promotion of developmentally appropriate sport. She also publishes in the area of coaching and coach education.

**Ashley Stirling**, PhD, is an associate professor, teaching stream, and the vice-dean, Academic Affairs, in the Faculty of Kinesiology and Physical Education at the University of Toronto. Her research expertise is in the areas of coach education and athletes’ experiences of maltreatment in sport.

**Stephanie Buono**, PhD, from the Department of Applied Psychology & Human Development at the University of Toronto, is currently working as a research associate. Her areas of research include emotional development, emotion regulation, and affective disorders. She has experience conducting statistical analyses to investigate the efficacy of emotion management strategies, and inform mental health and educational policy.
